# Supplementary material for: Resolving the full spectrum of human genome variation using Linked-Reads
Source: Genome Res. 2019 Apr;29(4):635–45. doi: 10.1101/gr.234443.118 (PMC6442396; doi:10.1101/gr.234443.118)
Supplement: Supplemental Material [file supp_gr.234443.118_Supplemental_longranger-2.2.zip › longranger-2.2.tar.bz2!/lr2.2-public/lib/stan/cmdstan/stan/doc/api/html/functions_vars_t.html]

Stan: Class Members - Variables


|  |  |
| --- | --- |
|  | Stan  2.5.0  probability, sampling & optimization |


- Main Page
- Related Pages
- Namespaces
- Classes
- Files

- Class List
- Class Index
- Class Hierarchy
- Class Members

- All
- Functions
- Variables
- Typedefs
- Enumerator
- Related Functions

- \_
- a
- b
- c
- d
- e
- f
- g
- h
- i
- k
- l
- m
- n
- o
- p
- q
- r
- s
- t
- u
- v
- w
- x
- y
- z


All Classes Namespaces Files Functions Variables Typedefs Enumerations Enumerator Friends Macros Pages

### - t -

- t0\_
  : stan::gm::integrate\_ode
  , stan::mcmc::stepsize\_adaptation
- T\_
  : stan::mcmc::base\_static\_hmc< M, P, H, I, BaseRNG >
- term\_g
  : stan::gm::expression07\_grammar< Iterator >
- term\_r
  : stan::gm::term\_grammar< Iterator >
- theta\_
  : stan::gm::integrate\_ode
  , stan::math::coupled\_ode\_system< F, double, stan::agrad::var >
  , stan::math::coupled\_ode\_system< F, stan::agrad::var, stan::agrad::var >
- theta\_dbl\_
  : stan::math::coupled\_ode\_system< F, double, double >
  , stan::math::coupled\_ode\_system< F, double, stan::agrad::var >
  , stan::math::coupled\_ode\_system< F, stan::agrad::var, double >
  , stan::math::coupled\_ode\_system< F, stan::agrad::var, stan::agrad::var >
- thin
  : stan::io::stan\_csv\_metadata
- timing
  : stan::io::stan\_csv
- tinyness\_before
  : std::numeric\_limits< stan::agrad::fvar< T > >
  , std::numeric\_limits< stan::agrad::var >
- tolAbsF
  : stan::optimization::ConvergenceOptions< Scalar >
- tolAbsGrad
  : stan::optimization::ConvergenceOptions< Scalar >
- tolAbsX
  : stan::optimization::ConvergenceOptions< Scalar >
- tolRelF
  : stan::optimization::ConvergenceOptions< Scalar >
- tolRelGrad
  : stan::optimization::ConvergenceOptions< Scalar >
- traps
  : std::numeric\_limits< stan::agrad::fvar< T > >
  , std::numeric\_limits< stan::agrad::var >
- truncation\_
  : stan::gm::sample
- truncation\_range\_r
  : stan::gm::statement\_grammar< Iterator >
- ts\_
  : stan::gm::integrate\_ode
- type\_
  : stan::gm::array\_literal
  , stan::gm::binary\_op
  , stan::gm::double\_literal
  , stan::gm::fun
  , stan::gm::index\_op
  , stan::gm::int\_literal
  , stan::gm::unary\_op
  , stan::gm::variable
- type\_identifier\_r
  : stan::gm::bare\_type\_grammar< Iterator >


---

[ Stan Home Page ]

*© 2011–2014,
Stan Development Team.*
